# Eye, Ear, Nose and Throat

**Published:** 1893-09

**Authors:** 


### EYE, EAR, NOSE AND THROAT.

EDITED BY DR. ARTHUR G. HOBBS, ASSISTED BY DR. GEORGE BROWN.

### A NEW METHOD OF MAKING A DIFFERENTIAL DIAGNOSIS BETWEEN LABYRINTHINE AND MIDDLE EAR DISEASE.

Dr. Ludwig Jankan, of Zurich, presents in a preliminary communication the following means of determining whether the labyrinth or the middle ear is the seat of disease in certain cases of deafness: In an observation of the phenomena in one hundred and four cases of ear disease, sixty-eight of which were affected in sound-conducting apparatus, and thirty-six in the sound-perceiving apparatus, he discovered that in cases of disease of the conducting apparatus, sound was conducted to his ear by means of a stethoscope connected with the patent's ear more loudly by the more affected ear, while the less affected ear in disease of the sound-perceiving organ heard the sound more loudly from the less affected ear.—*American Journal of Medical Sciences.*

[Should these experiments be confirmed, the method would prove to be a valuable one, especially when a doubt should exist after all other and simpler means had been resorted to. It would be too impractical for constant clinical use.—ED.]

### OPHTHALMOPHASMATOSCOPY.

Dr. Alex. Trantas has devised a method of making a spectroscopic examination of the contents of the eye, in regard to which he makes the following remarks: The eye is the only organ of the human body, the media of which we can submit to a spectroscopical examination. If in proceeding as for an ophthalmoscopic examination, in place of receiving the rays from the eye of the observed ourselves, we make them pass through a spectroscope, it is evident that we would have different spectral lines, according to the difference in the media of the eye. For example: If there existed hemorrhage in the vitreous, we would see the special spectrum for this substance, and by this very sensitive means we could determine whether the flecks in the vitreous body were blood or exudate. It is well known that there are cases where it is absolutely impossible to determine this distinction by the means known to science, because very minute quantities of blood escape ophthalmoscopical examination. In such a case the spectroscope could also discover for us the pathological condition. But could not the changes which the vitreous and other media of the eye undergo, because of different diseases of the eye, and those caused by change in general nutrition (toxines, etc.), be interpreted by the different spectra peculiar to each case? Dr. Trantas is making researches with this object in view.—*Archives d'Opthalmologie, Occidental Med. Times.*

[If Dr. Trantas's future experiments should confirm those already made, and if his spectroscopic examinations can be made clinically practical, a great advance will have been made in the differential diagnoses of vitreous opacities.—ED.]

---

### GLAUCOMA.

Mr. Henry Juler (*Clinical Journal,* December 21, 1892) expresses the opinion that a large upward iridectomy is the only effective method of relieving abnormal increase of tension. Eserine and pilocarpine have the effect of reducing increased tension, but they are tolerated for a short time only and soon lose their effects. Where operation must be postponed instill eserine and cocaine, apply leeches to the temple, hot fomentations, etc., and give a suitable aperient. In subacute cases this line of treatment should be carried out. In chronic cases use eserine and keep the patient under careful observation. In acute cases operate as soon as possible. In subacute cases operation is justifiable as long as there is perception of light. Excellent results in improved vision and freedom from pain may be obtained. In chronic cases where the contraction of the visual field has not passed the center, an operation should be made. If contraction has extended beyond this, it is a question whether the eye should not be left to nature, as after prolonged tension the tissues are weakened, and such eyes stand operations badly.—*International Medical Magazine.*

### COMBINATION OF SEVERAL ALKALOIDS IN A SOLUTION FOR USE AS A COLLYRIUM.

Dr. Berger thinks by a combination of alkaloids we get a more active, while at the same time less poisonous, preparation for use in the eyes. By the use of a mixture of equal parts of one per cent. solutions of atropine, duboisine and cocaine we obtain a mydriatic action unobtainable by any other means. A mixture of

Sulph. atropine, } aa 30 c. grms.

" duboisine, }

Chlorhyd. cocaine, 2 grms.

Dist. water, 100 grms.

is a mydriatic at least as powerful as a 1 per cent. solution of atropine, without being as toxic. A mixture of

Sulph. eserine, 1 grms.
Hydrochlor. pilocarpine, 2 grms.
Dist. water, 100 grms.

is very efficacious and well supported. A mixture as follows:

Chlorhydrate cocaine, } aa. 2 grms.

" pilocarpine, }

Dist. water, 100 grms.

presents all the qualities of cocaine with the further advantage, that the mydriasis and the difficulties of accommodation observed in the eye simply cocainized, are wanting.—*Rev. de Therap. Times and Register.*

---

### ILLUMINATION OF THE EYES FROM BEHIND.

When an incandescent lamp is introduced into the mouth, for the purpose of ascertaining, by trans-illumination, the condition of the maxillary antra, the pupils are seen as blood-red apertures. Mr. N. Stevenson calls attention to the curious fact that when the light is thus introduced, as it were, by a back door, the pupils do not contract. This observation may prove to possess considerable importance.—*The Medical Press.*

